# Differential Effects of Surface-Functionalized Zirconium Oxide Nanoparticles on Alveolar Macrophages, Rat Lung, and a Mouse Allergy Model

**DOI:** 10.3390/nano7090280

**Published:** 2017-09-19

**Authors:** Antje Vennemann, Francesca Alessandrini, Martin Wiemann

**Affiliations:** 1 IBE R&D Institute for Lung Health gGmbH, Mendelstr. 11, 48149 Münster, Germany; vennemann@ibe-ms.de; 2 Center of Allergy and Environment (ZAUM), Technical University of Munich and Helmholtz Center Munich, Member of the German Center for Lung Research (DZL), Ingolstädter Landstr. 1, 85764 Neuherberg, Germany; franci@helmholtz-muenchen.de

**Keywords:** macrophage model, intratracheal administration, ZrO_2_ nanoparticles, inflammation, surface labelling, polyethylene glycol, allergy

## Abstract

Nanoparticles (NPs) may affect the lung via their chemical composition on the surface. Here, we compared the bioactivity of zirconium oxide (ZrO_2_) NPs coated with either aminopropilsilane (APTS), tetraoxidecanoic acid (TODS), polyethyleneglycol (PGA), or acrylic acid (Acryl). Supernatants from NPs-treated cultured alveolar macrophages (NR8383) tested for lactate dehydrogenase, glucuronidase, tumor necrosis factor α, and H_2_O_2_ formation revealed dose-dependent effects, with only gradual differences among particles whose gravitational settling and cellular uptake were similar. We selected TODS- and Acryl-coated NPs for intratracheal administration into the rat lung. Darkfield and hyperspectral microscopy combined with immunocytochemistry showed that both NPs qualities accumulate mainly within the alveolar macrophage compartment, although minute amounts also occurred in neutrophilic granulocytes. Dose-dependent signs of inflammation were found in the broncho-alveolar lavage fluid on day 3 but no longer on day 21 post-application of ≥1.2 mg per lung; again only minor differences occurred between TODS- and Acryl-coated NPs. In contrast, the response of allergic mice was overall higher compared to control mice and dependent on the surface modification. Increases in eosinophils, lymphocytes and macrophages were highest following ZrO_2_-PGA administration, followed by ZrO_2_-Acryl, ZrO_2_-TODS, and ZrO_2_-APTS. We conclude that surface functionalization of ZrO_2_ NPs has minor effects on the inflammatory lung response of rats and mice, but is most relevant for an allergic mouse model. Allergic individuals may therefore be more susceptible to exposure to NPs with specific surface modifications.

## 1. Introduction

The inhalation of airborne nanomaterials may lead to adverse health effects in the lung such as inflammation, granule formation, or even genotoxic effects [1,2,3]. Inhaled NPs deposited in the lung parenchyma may primarily interact with biomolecules of the lung surfactant and the lung lining fluid. Chemical properties of the NPs surface, such as surface charge and hydrophobicity, likely determine the composition of the protein and/or lipid corona of nanoparticles in a biological environment [4,5,6] and this, in turn, may influence subsequent biological responses such as the uptake into phagocytic cells, permeation of the lung barrier, or transport via draining lymphatic vessels [7]. The chemical nature of the NPs surface may, therefore, indirectly influence the biodistribution and effects of NPs in the body.

Effects of bioactive or toxic NPs have already been shown to be modified by surface coating. For example, various coatings of colloidal silica NPs with a mean size of 15 nm can influence the inflammatory properties of the core material both in vitro and in vivo [8,9,10]. While the pristine, i.e., uncoated material elicited inflammatory effects upon inhalation and administration, coating with phosphonate, amine, or polyethylene glycol residues gradually abrogated cytotoxic and inflammogenic effects in the rat lung [9,10]. Interestingly, phosphonate or silica coating also mitigated the toxic effects of other metal oxide particles [11,12], suggesting that a chemical shielding of the nanoparticle surface can downregulate its toxic or inflammatory effects.

However, the question remains as to whether chemical surface modifications or functionalization have effects on their own and may become apparent for NPs with low specific toxicity. In this context, ZrO_2_ NPs are of pivotal interest. These NPs are industrially relevant, e.g., as self-cleaning coating of stoves, as part of dental composites, or in other biomedical applications [13]. However, for an appropriate embedding into organic matrices, ZrO_2_ NPs often need stable polymer coatings, two of which have already been tested for possible effects on the lung or on cell models [14]. Thus, ZrO_2_ nanoparticles coated with tetraoxadecanoic acid or acrylic acid, which are also used in this study, elicited no inflammatory or toxic responses within 21 days post-application in rat lungs, at least upon a lung burden of up to 600 μg achieved with a short-term inhalation protocol [9]. 

In this work we aimed to compare the effects of specific surface coatings of ZrO_2_ NPs using four different industrially relevant coatings frequently used to enhance particle dispersion, namely basic aminopropylsilane (APTS), acidic tetraoxadecanoic acid (TODS), and acidic acrylic acid (Acryl) coating [15]. Furthermore, a polyethyleneglycol (PGA) coating was enrolled in the study, which in many applications confers on nanoparticles a considerable degree of biocompatibility, i.e., by preventing their rapid uptake by the reticuloenthothelial system [16,17,18,19]. Importantly, the four types of ZrO_2_ NPs had the same size (9–10 nm) and were provided as stable suspensions, such that extensive ultrasonic treatment, often required to disperse powder nanomaterials, was not necessary. In the study we used three different models: (i) an in vitro model of pulmonary toxicity using alveolar macrophages, (ii) an in vivo toxicity model in the rat lung, and (iii) the mouse lung allergy model, in which effects on allergic animals are compared with non-treated control animals [20]. 

Experiments with alveolar macrophages appear highly adequate to predict the lung toxicity of NPs, as these cells are among the first to accumulate NPs in the lung. From in vitro tests NR8383 alveolar macrophages from rats were shown to be of high predictive value for rat experiments because a combined evaluation of four parameters, namely lactate dehydrogenase (LDH), glucuronidase (GLU), tumor necrosis factor α (TNF α), and H_2_O_2_ formation was sufficient to predict lung toxicity of nanoparticles, at least for 19 nanomaterials tested so far in short-term inhalation studies [8]. The test was used to select two ZrO_2_ NPs for further in vivo testing.

The administration of particles into the rat lung followed by the analysis of the bronchoalveolar lavage fluid (BALF) after three and 21 days was then conducted to further describe inflammatory and other short-term effects. The dose range used in this study was deduced from the in vitro results in such a way that it spans from the no-adverse-effect level up to effective doses that are always necessary to demonstrate and compare particle surface-dependent effects. 

Finally, we used a mouse allergy model, thus adding another susceptibility factor to the study. Allergic animals have been shown to respond in a more sensitive manner to a given particle burden of the lung [20], and oxidative stress seems to play an important role in particle-induced exacerbation of lung allergic inflammation [21]. While early allergic responses in the respiratory tract appear to be mediated by an IgE-mediated release of inflammatory substances from mast cells and basophils, the late-phase reaction following local allergen challenge is characterized by recruitment, activation, and infiltration of leucocyte populations [22]. In a previous study we successfully used BALF cell analysis to differentiate between adjuvant effects of silica NPs with different coating agents [10]. 

Our study shows that the coating of ZrO_2_ NPs with APTS, TODS, Acryl, or PGA has little influence on the bioactivity of ZrO_2_ NPs in the macrophage assay or the rat lung. On the contrary, allergic animals respond in a more sensitive manner to specific surface coatings of ZrO_2_ NPs.

## 2. Results

### 2.1. Particle Behavior under Cell Culture Conditions

**Figure 1:**
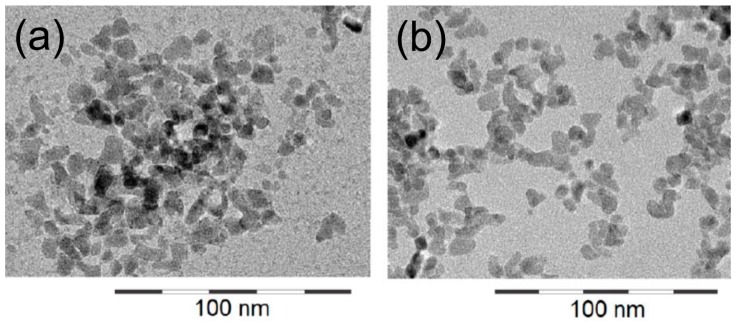
Transmission electron microscopic images of ZrO_2_-TODS (**a**) and ZrO_2_-Acryl (**b**). Images were taken from a previous characterization study [24].

**Figure 2:**
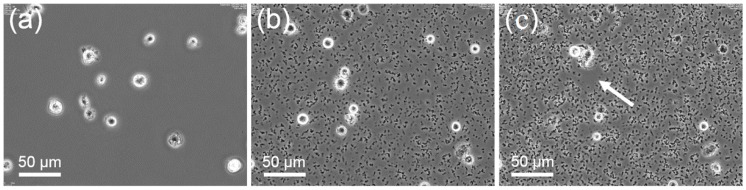
Progressive gravitational settling of ZrO_2_-TODS in the presence of NR8383 cells. Phase contrast images were taken from a time-lapse observation series 3 min (**a**), 75 min (**b**), and 150 min (**c**) after addition of particles (11.25 μg/mL). A moving NR8383 cell has just cleared a small area (arrow in **c**) from particles.

The primary size of all nanoparticles used in this study was 9–10 nm, as shown by electron microscopy (Figure 1). However, according to their specific properties (Table S1) which have been previously investigated [23,24]), ZrO_2_ NP formed agglomerates upon dilution in serum-free F-12K cell culture medium or glucose-containing phosphate-buffered Krebs Ringer (KRPG). Agglomerates were visible by phase contrast microscopy and accumulated at the bottom of the cell culture vessel, as shown in Figure 2. The time course of gravitational settling of all four types of particle has been previously described and follows hyperbolic functions [25]. Time constants estimated from these sedimentation curves of particles amounted to 70, 87, 132, and 160 min for ZrO_2_-TODS, ZrO_2_-PGA, ZrO_2_-APTS, and ZrO_2_-Acryl, respectively. Accordingly the sedimentation of particle agglomerates was complete within the cell culture test period of 16 h. At this time point optical particle tracking of the F-12K cell culture medium containing 180 μg/mL of either particle quality also failed to show nano-objects above the detection limit (<10^7^ particles/mL). Together the experiments show that all ZrO_2_ NPs form loose agglomerates and undergo complete gravitational settling.

### 2.2. In Vitro Results with NR8383 Alveolar Macrophages

**Figure 3:**
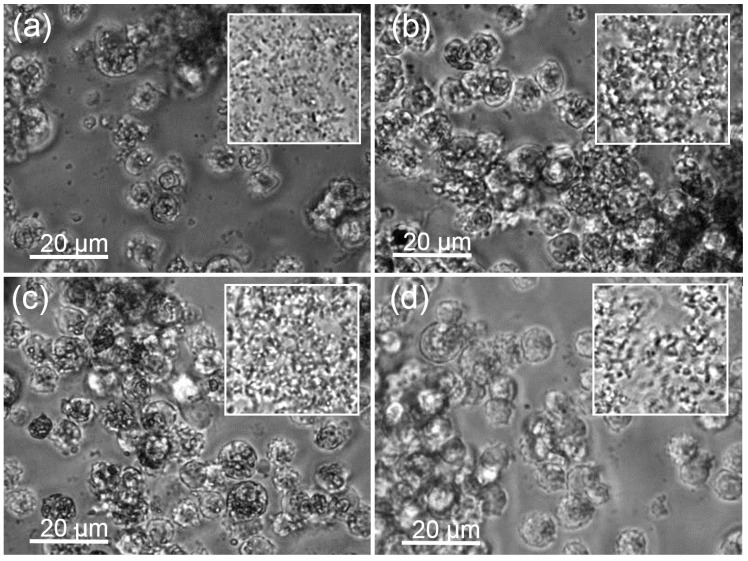
Uptake of ZrO_2_ particle agglomerates by alveolar macrophages under testing conditions. (**a**) ZrO_2_-Acryl, (**b**) ZrO_2_-TODS, (**c**) ZrO_2_-APTS, and (**d**) ZrO_2_-PGA. Cultures of 3 × 10^5^ cells per well of a 96-well plate were laden with differently coated ZrO_2_ NPs (90 μg/mL), as indicated and incubated for 16 h. Insets show particles settled under identical conditions in the absence of cells. Note that the space between cells is largely cleared from particles and that cells contain dark material.

Sedimented particles were cleared nearly completely from the bottom of the culture vessel by NR8383 macrophages up to the maximum concentrations applied after 16 h (Figure 3). Macrophages filled with engulfed material appeared darker when viewed with phase contrast optics. Deteriorated cells were observed at higher concentrations of all ZrO_2_ NPs.

**Figure 4:**
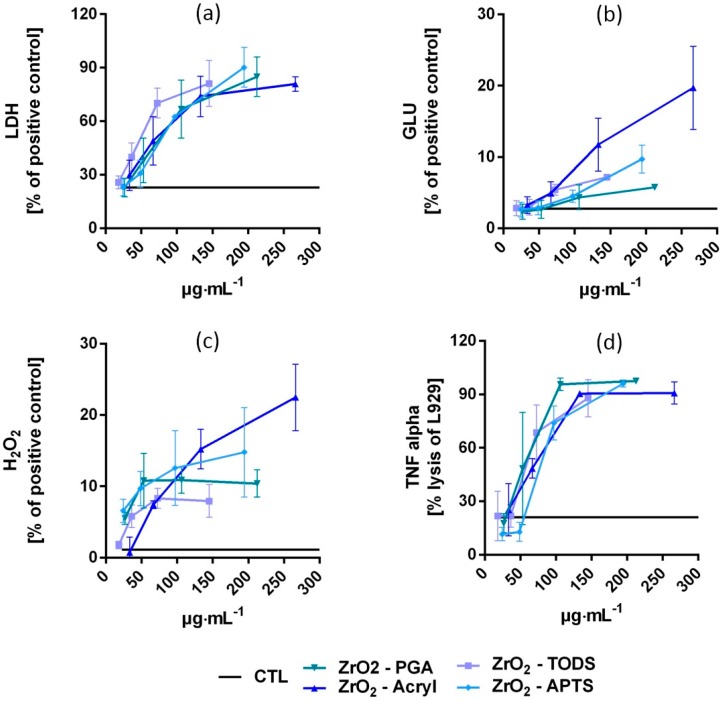
Dose-dependent effects of the four differently coated ZrO_2_ NPs on NR8383 alveolar macrophages in vitro. Cultures of 3 × 10^5^ cells per well of a 96-well plate were laden with either ZrO_2_-Acryl, ZrO_2_-APTS, ZrO_2_-TODS, or ZrO_2_-PGA and incubated for 16 h. Culture supernatants were assessed for (**a**) activity of lactate dehydrogenase (LDH) in percent of triton X-100 lysed control cells, (**b**) activity of glucuronidase (GLU) in percent of triton X-100 lysed control cells, (**c**) extracellular H_2_O_2_ in percent of the zymosan-treated positive control, and (**d**) tumor necrosis factor α (TNF alpha), as indicated by the lysis of L929 fibroblasts. Values are means ± SD from three independent experiments; Level of untreated controls (CTL) is indicated by solid lines; concentration values were corrected according to post hoc gravimetric measurements (see Methods section). Values from ZrO_2_-Acryl and ZrO_2_-TODS were taken from [8].

All four types of ZrO_2_ NPs had dose-dependent effects on NR8383 alveolar macrophages in vitro, as shown by the curves in Figure 4 and the half maximal effective concentration (EC50) values in Table 1. The degree of cytotoxicity, indicated by the release of LDH, was similar for ZrO_2_-Acryl, ZrO_2_-APTS, and ZrO_2_-PGA, whereas ZrO_2_-TODS was slightly more cytotoxic. Dose–response curves and EC50 values were also similar for the release of bioactive TNFα, as measured by the lysis of L929 fibroblasts. Release of the lytic enzyme glucuronidase, which reflects macrophage activation and/or damage of phagosomes, appeared to be more differentiated, with the rank order being ZrO_2_-Acryl > ZrO_2_-TODS ≥ ZrO_2_-APTS > ZrO_2_-PGA. Release of H_2_O_2_ into the extracellular space was moderate and only slight differences between particles were seen. Depending on the slope of the curves the rank order of H_2_O_2_ release was ZrO_2_-Acryl ≥ ZrO_2_-APTS ≥ ZrO_2_-PGA ≥ ZrO_2_-TODS. Importantly, the particle-free supernatant prepared by centrifugation from all particle preparations elicited no changes in LDH, GLU, TNF, or H_2_O_2_ (Figure S1). In vitro effects can, therefore, be ascribed to particles settled onto and/or engulfed by NR8383 macrophages and are not due to dissolved substances. 

### 2.3. In Vivo Study in the Rat Model

Based on the in vitro test results, we selected ZrO_2_-TODS and ZrO_2_-Acryl for further in vivo testing, as overall analysis revealed the largest differences between these types of NPs. Aiming to include the no-adverse-effect level in the three treated groups, we estimated an appropriate dose range from the in vitro results: as shown in Figure 4a, the threshold for cytotoxic effects in vitro is in the range of 22.5–45 μg/mL. As the uptake of particles by a defined number of cells per well was complete, a concentration of 22.5–45 μg/mL equals a mean cellular dose of 15–30 pg/cell. Multiplying this value by the typical number of ~2 × 10^7^ alveolar macrophages per rat lung [26] suggested that a lung dose of 30 × 2 × 10^7^ pg (i.e., 600 μg) may be fully cleared by macrophages and evokes no further damage in the lung. The study was, therefore, conducted with 0.6, 1.2 and 2.4 mg ZrO_2_-TODS and ZrO_2_-Acryl per rat lung with two follow-up periods of three and 21 days. 

#### 2.3.1. Localization and Cellular Distribution of ZrO_2_ Nanoparticles 

**Figure 5:**
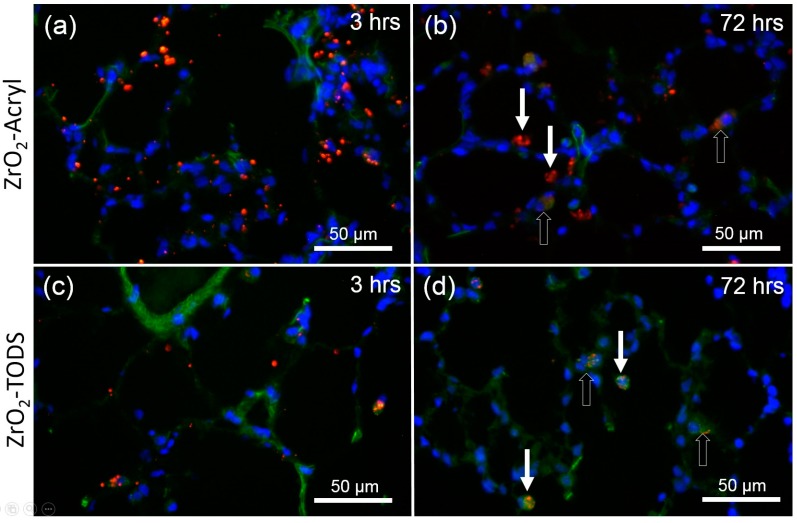
Early distribution of ZrO_2_ NPs in the lung. Rat lungs were dosed with 1.2 mg of fluorescent ZrO_2_-Acryl (**a**,**b**) or ZrO_2_-TODS (**c**,**d**) and sections were evaluated after 3 h (**a**,**c**) and three days (**b**,**d**). Particles (red) were labeled with Texas Red NHS covalently linked to a protein corona of rat serum albumin adsorbed to nanoparticles prior to administration. Cell nuclei are stained blue with 4′,6-diamidino-2-phenylindole (DAPI). Alveolar tissue components show green autofluorescence upon illumination with blue light (488 nm). Note that 3 h post-administration, numerous fluorescent particles or agglomerates line the alveolar septae (**a**,**c**). After three days, red fluorescence is concentrated in phagocytic cells (**b**,**d**) located in alveoli or in lung epithelium (open arrows).

ZrO_2_ NPs are translucent particles that are hard to identify in unstained tissue by conventional light microscopy. However, as particle dispersion was achieved by albumin coating, we covalently labeled the resultant protein corona with TexasRed^®^ succinimidyl ester and delivered 1.2 mg of the fluorescent particles into the lungs of two satellite animals. Fluorescence microscopy of lung cryo-sections prepared 3 h post-administration showed a multitude of fine fluorescent particles, mostly adhering to or colocalized with alveolar septa, which were visible as green, autofluorescent structures (Figure 5a,c). Early uptake into phagocytic cells was occasionally noted. After three days alveolar septa were cleared from red fluorescent particles, which occurred nearly exclusively in single phagocytic cells located inside the alveolar space or within the alveolar septa (Figure 5b,d). No fluorescent NPs were detected in 4′,6-diamidino-2-phenylindole(DAPI)-stained nuclei and no major difference was found between ZrO_2_-Acryl and ZrO_2_-TODS with respect to their early distribution pattern.

**Figure 6:**
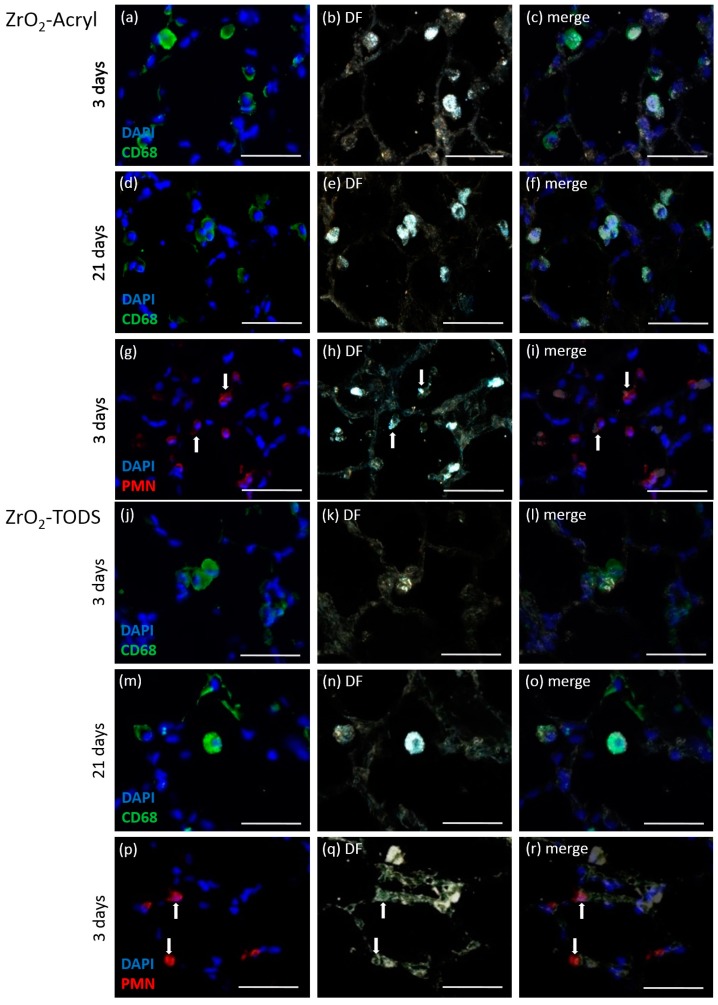
Identification of ZrO_2_-Acryl- and ZrO_2_-TODS- containing lung cells. Immunocytochemical detection of macrophages and polymorphonuclear leukocytes (PMN) was combined and merged with dark field (DF) images. Sections were from rat lungs dosed with 2.4 mg ZrO_2_-Acryl (**a**–**i**) or ZrO_2_-TODS (**j**–**r**) and evaluated after three days (**a**–**c**,**g**–**i**,**i**–**l**,**p**–**r**) and 21 days (**d**–**f**,**m**–**o**). CD68-positive alveolar macrophages show green fluorescence (**a**,**d** and **j**,**m**), PMN are immunolabeled in red (**g**–**i**,**p**–**r**). Cell nuclei are stained blue with 4′,6-diamidino-2-phenylindole (DAPI). Dark field images (**b**,**e**,**h** and **k**,**n**,**q**) show light scattering material concentrated in CD68 positive alveolar macrophages (**c**,**f** and **l**,**o**), or, to lower extent, in some PMN (**i**,**r**), as indicated by white arrows. Bars: 50 µm.

To identify particle-laden phagocytic cells, we immuno-labeled alveolar macrophages as well as polymorphonuclear leukocytes (PMN). The sections were also viewed with enhanced dark field microscopy (DF) to look for the distribution of light-scattering particulate material. As shown for the high concentration group exposed to 2.4 mg of either ZrO_2_-Acryl or ZrO_2_-TODS (Figure 6), alveolar macrophages, positive for the CD68 protein, contained large amounts of light-scattering material on day 3, even more pronounced on day 21. PMN were frequently found in particle-laden areas of the lung tissue on day 3 and also occasionally contained low amounts of light-scattering material. Together these studies show that ZrO_2_-Acryl and ZrO_2_-TODS delivered into the lung as a nano-dispersion (Figure S2) were progressively collected and engulfed by alveolar macrophages and to a far lower degree by PMN, whereas no indications were found of an uptake into the lung epithelium.

#### 2.3.2. Analyses of the Broncho-Alveolar Lavage Fluid (BALF)

**Figure 7:**
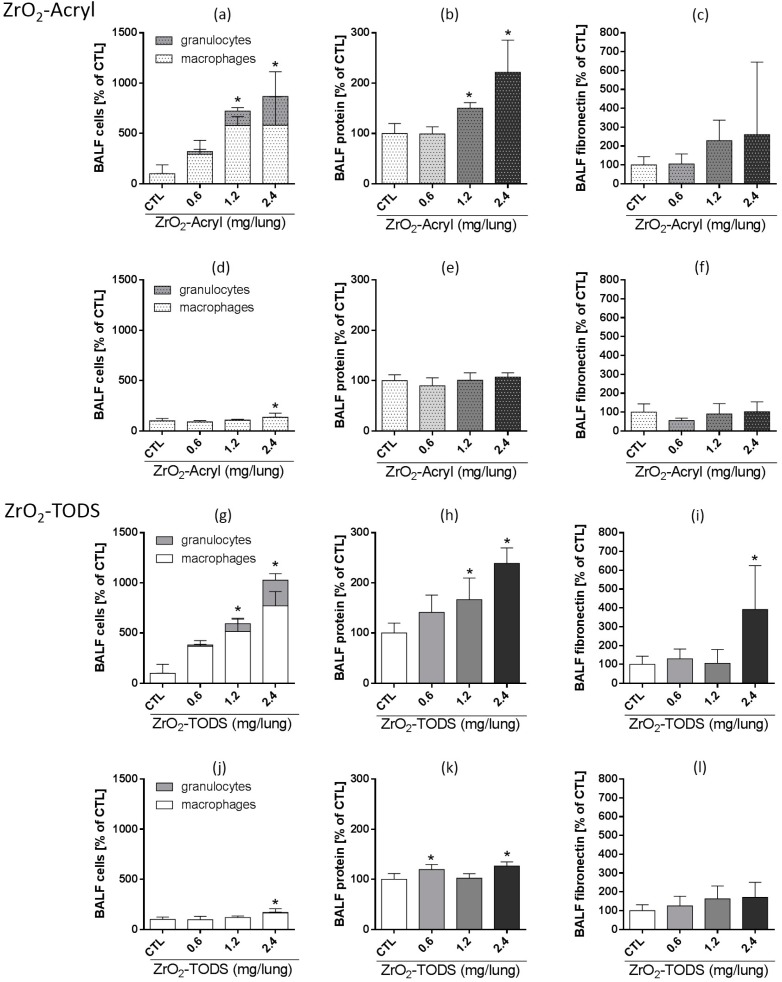
Effects of ZrO_2_-Acryl and ZrO_2_-TODS on the rat lung. Indicated doses of ZrO_2_-Acryl were intratracheally delivered and effects on BALF parameters are shown as percent vehicle control at three (**a**–**c**,**g**–**i**), and 21 days (**d**–**f**,**j**–**l**) post-administration. (**a**,**d** and **g**,**j**) Differential cell counts reveal alveolar macrophages and neutrophilic granulocytes as the dominant cell types. (**b**,**e** and **h**,**k**) Protein concentration as measured with the Lowry method. (**c**,**f** and **i**,**l**) Fibronectin was quantified by a specific enzyme-linke immunosorbent assay (ELISA). Values are mean ± SD from *n* = 5 rats; * *p* < 0.05 was revealed by one-way analysis of variance (ANOVA) followed by Dunnett’s multiple comparison test.

To better quantify and compare the inflammatory effects of intratracheally administered ZrO_2_-Acryl and ZrO_2_-TODS NPs, we analyzed the bronchoalveolar lavage fluid (BALF) from all groups. We found dose-dependent signs of inflammation not seen in BALF from control animals that received the vehicle only. Again, there were only minor differences between the types of particles: both ZrO_2_-Acryl and ZrO_2_-TODS (Figure 7) led to dose-dependent increases in the number of total cells in BALF, mainly composed of macrophages and PMN, as well as an increased concentration of total BALF protein three days post-administration. For both types of particles, effects reached the level of significance at ≥1.2 mg per lung. Fibronectin, which is an indicator of extracellular matrix formation, increased upon administration of 2.4 mg ZrO_2_-TODS per lung; there was a similar tendency for ZrO_2_-Acryl, which failed to reach a level of significance due to some variability. All inflammatory changes in BALF were reversible and decayed down to the control level after 21 days, except for minor increases in macrophage number and protein concentration upon administration of 2.4 mg of either ZrO_2_-TODS or ZrO_2_-Acryl.

**Figure 8:**
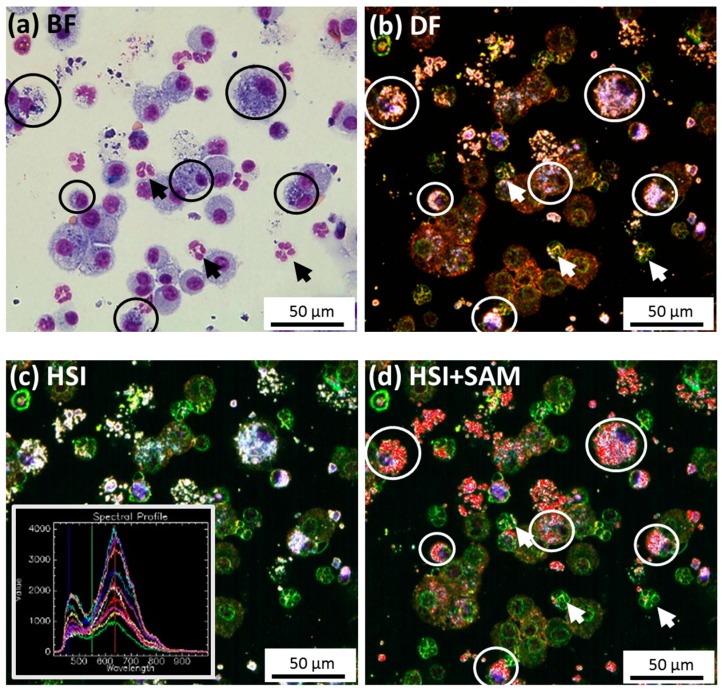

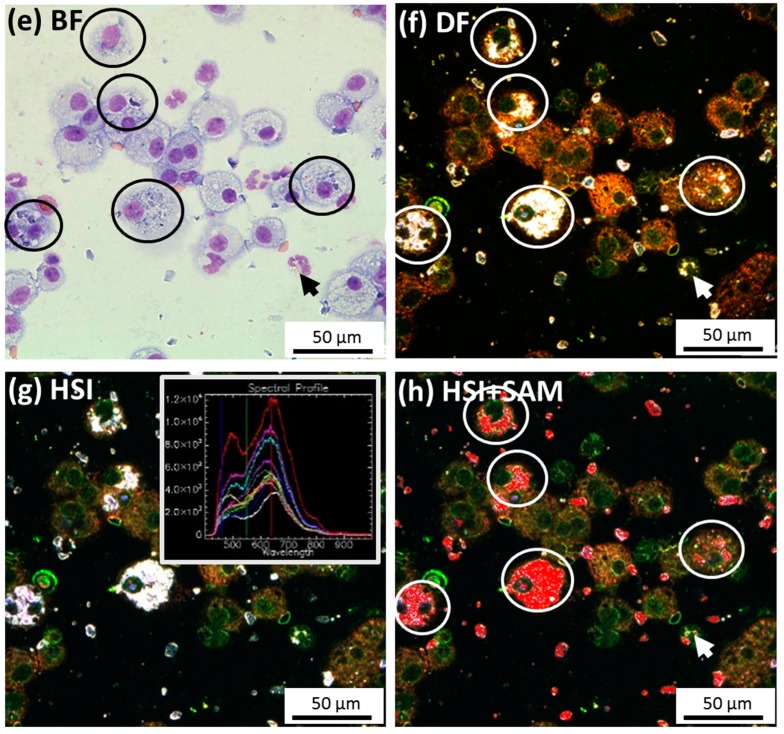
Identification of ZrO_2_-Acryl NPs and ZrO_2_-TODS in Pappenheim-stained cytospin preparations from rat BALF. Rats were administered 2.4 mg ZrO_2_-Acryl (**a**–**d**) or ZrO_2_-TODS NPs per lung (**e**–**h**) and lavaged three days post-administration. (**a**,**e**) Bright field images showing alveolar macrophages (encircled) and granulocytes. (**b**,**f**) Corresponding dark field images; light scattering material is contained in alveolar macrophages. (**c**,**g**) Corresponding pseudo-color HSI images; the spectral libraries for ZrO_2_-Acryl and ZrO_2_-TODS (insets) were collected from encircled macrophages laden with light-scattering material. (**d**,**h**) Matching of the spectral libraries from (**c**,**g**) with all data points according to the spectral angle mapping (SAM) method. ZrO_2_-Acryl- and ZrO_2_-TODS-positive pixels (superimposed in red) occur in macrophages and to a lesser extent in some granulocytes (arrows in **a**,**b**,**d** and **e**,**f**,**h**).

The finding that PMN contained light-scattering material in tissue sections prompted us to closer analyze cells in cytospin preparations for their particle content. To this end, we employed hyperspectral imaging (HSI) which uses the spectral information from light-scattering structures such as nanoparticles to identify identical materials. Analyses of both materials are shown in Figure 8: compared to Pappenheim’s staining, where particle inclusions in macrophages appear as dark regions in the cytoplasm (Figure 8a,e), the same structures appear as bright white inclusions in the DF image and also in the pseudo color HSI image. Assuming that these bright inclusions within macrophages represent (agglomerated) ZrO_2_ NPs, as they were not found in the controls, we composed spectral libraries from these structures (see insets in Figure 8). Application of these libraries to the complete images, along with the “Spectral Angle Mapper” (SAM) method, matched nearly all of the light-scattering material inside and outside cells, suggesting this material chiefly contains ZrO_2_-Acryl or ZrO_2_-TODS. In this way several PMN (arrows in Figure 8d,h) were identified to contain small inclusions whose hyperspectrum was identical to that of inclusions in macrophages. 

### 2.4. In Vivo Study in the Allergy Mouse Model

In this part of the study we compared the effects of ZrO_2_-APTS, ZrO_2_-TODS, ZrO_2_-Acryl, and ZrO_2_-PGA in a mouse allergy model compared to healthy animals. Based on the threshold dose of 0.6 to 1.2 mg observed for the rat lung, we used a single particle dose of 100 μg per mouse lung, which we expected to elicit low but significant effects in the more susceptible allergy model. 

**Figure 9:**
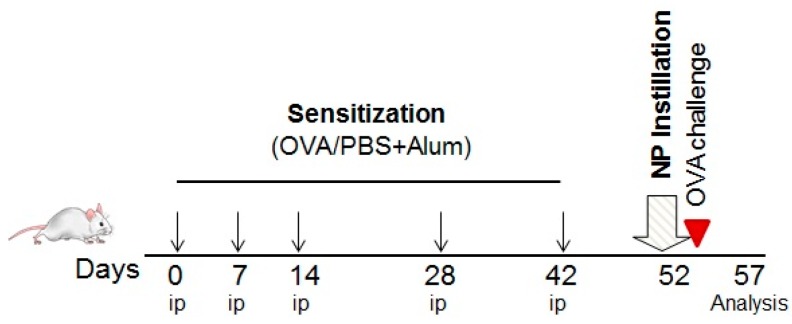
Experimental scheme for ovalbumin sensitization, NPs administration, and ovalbumin challenge. OVA, ovalbumin; PBS, Dulbecco’s phosphate buffer saline; Alum, aluminum hydroxide; ip, intraperitoneal injection.

**Figure 10:**
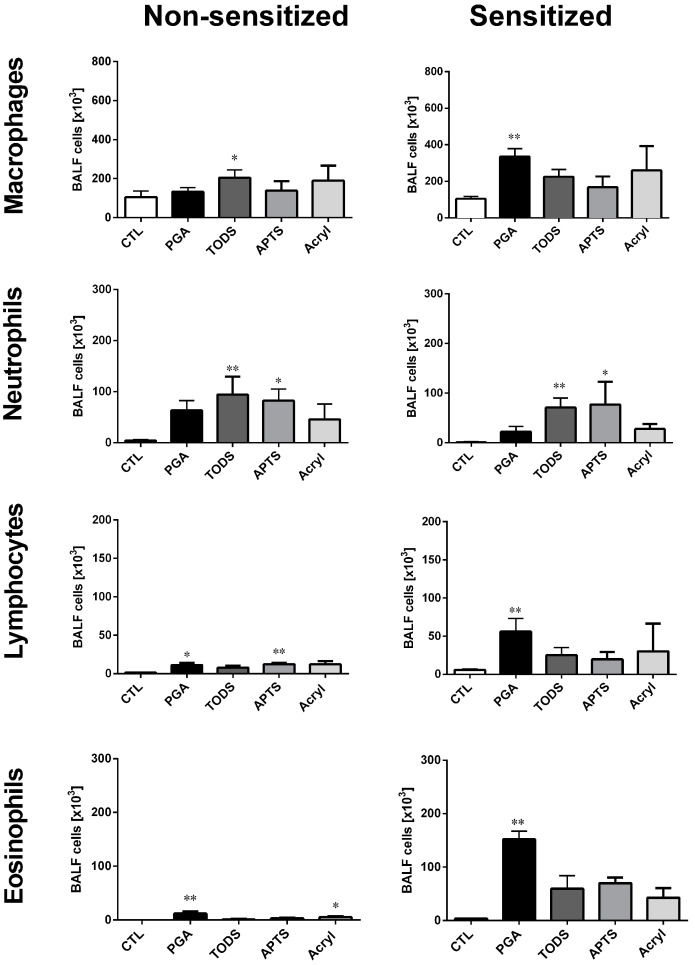
Effect of surface-modified ZrO_2_ NPs on bronchoalveolar lavage cells of ovalbumin-sensitized and non-sensitized mice. Each group of mice (*n* = 5) received 100 μg per mouse of ZrO_2_-APTS, ZrO_2_-TODS, ZrO_2_-Acryl, or ZrO_2_-PGA prior to OVA challenge. CTL: supernatant-treated controls. * *p* < 0.05; ** *p* < 0.01 vs. CTL was revealed by one-way ANOVA followed by Kruskal-Wallis test.

Mice were sensitized to ovalbumin (OVA) according to the experimental protocol shown in Figure 9. Controls were sham-sensitized (PBS/Alum). On day 52 mice received a single intratracheal dose of ZrO_2_ NPs and all animals were subsequently challenged with ovalbumin after each inhalation_._ Ovalbumin challenge led to a slight increase in lymphocyte and eosinophil counts in the BALF from sensitized animals, whereas no effects were observed in the healthy control group. Administration of ZrO_2_-PGA, ZrO_2_-TODS, ZrO_2_-APTS, or ZrO_2_-Acryl prior to allergen challenge elicited specific effects in the BALF that differed in sensitized and non-sensitized animals. All particle effects on BALF cell populations from non-sensitized and sensitized animal are compared side-by-side in Figure 10.

The largest differential effects were observed for ZrO_2_-PGA, as it evoked a significant increase in both eosinophils and lymphocytes, which significantly exceeded the small though significant increases of these cell populations in non-sensitized animals. Also, macrophage numbers reacted differentially and a 3-fold increase was noted only in sensitized animals. In both groups there was a non-significant elevation of PMN. Results demonstrate the strongest adjuvant effect of PGA-coated ZrO_2_, compared to the other ZrO_2_ NPs, in allergic airway inflammation.

ZrO_2_-APTS, ZrO_2_-TODS, and ZrO_2_-Acryl also moderately increased BALF eosinophils and lymphocytes. Although the effects of all three NPs appeared to be more pronounced in sensitized than in non-sensitized mice, significance was not reached in the sensitized group. The numbers of PMNs were increased both in sensitized and in non-sensitized animals upon ZrO_2_-APTS and ZrO_2_-TODS but not ZrO_2_-Acryl administration. Macrophages were slightly increased following administration of all ZrO_2_ NPs, but, in addition to PGA in sensitized animals, only TODS led to significant effects in the non-sensitized group.

The rank order of the inflammatory potential based on the NPs-induced elevation of PMN counts in BALF is ZrO_2_-TODS > ZrO_2_-APTS > ZrO_2_-PGA > ZrO_2_-Acryl for the non-sensitized mice. The rank order of the adjuvant effect for allergic mice is ZrO_2_-PGA >> ZrO_2_-APTS ≥ ZrO_2_-TODS ≥ ZrO_2_-Acryl.

## 3. Discussion

In this study three well-established methods were used to investigate and compare the effects of four surface-functionalized ZrO_2_ nanoparticles. In vitro testing with alveolar macrophages (NR8383) showed that all particles, despite their different surface coating, which was most likely exposed to cells in a serum-free culture medium, underwent uniform gravitational settling. As uptake by alveolar macrophages was nearly complete, effects of different materials could be compared on the basis of equal cellular dose. What we observed upon particle exposure was a highly conserved pattern of the dose-dependent release of LDH, GLU, TNF*a*, and H_2_O_2_ with gradual differences, which prompted us to select ZrO_2_-TODS and ZrO_2_-Acryl as high- and low-toxicity variants, respectively, for subsequent intratracheal administration into the rat lung. Also, in this model, both materials elicited a similar transient inflammation, with ZrO_2_-TODS again being slightly more potent. In the mouse lung, where all four surface variants were tested, it was again ZrO_2_-TODS that evoked the highest numbers of PMN in BALF, indicative of lung inflammation. However, in the mouse allergy model, maximum increases of eosinophils, lymphocytes, PMN, and macrophages were caused by ZrO_2_-PGA, showing that sensitized animals react differently to the coating materials.

### 3.1. In Vitro Study of Alveolar Macrophages 

Compared to other metal oxide nanoparticles previously tested with the macrophage model, all surface modifications of ZrO_2_ were clearly more cytotoxic than, e.g., BaSO_4_, Fe_2_O_3_, and AlOOH, but were far less cytotoxic than, e.g., ZnO [8]. However, as measured by LDH release, they were slightly more cytotoxic than several types of CeO_2_, or TiO_2_ NM105. In contrast, the release of glucuronidase and TNFα, indicating macrophage activation [27,28], was smaller than observed, e.g., for TiO_2_ or CeO_2_ NPs, whereas the production of H_2_O_2_ was larger. Thus, the effects of metal oxides are not uniform and especially ZrO_2_ NPs show a tendency to increase cellular ROS production. Interestingly, this finding is in line with a recent study on PC12 and NA2 cells, demonstrating that uncoated ZrO_2_ in a similar dose range (5–50 nm) induces oxidative stress and also genotoxic effects [13]. Although that study was conducted in the presence of fetal calf serum and the uptake efficacy was not documented, it is interesting to note that oxidative effects commenced at a concentration of 31 μg/mL, which is close to the EC50 value of H_2_O_2_ release seen for ZrO_2_-TODS treatment. However, the effects of three surface coated ZrO_2_ NPs on ROS generation and cytotoxicity were not found in a study using 10 different cell lines [29] exposed to lower concentrations of 10 μg/cm^2^ in the presence of serum (equivalent to approximately 20 μg/mL). Together these findings suggest that ZrO_2_ NPs have some oxidative potential at elevated concentration that is not confined to macrophages. With respect to the differences caused by TODS, Acryl, PGA, and APTS coatings, as estimated from Figure 4 and the EC50 values of Table 1, disparities between minimum and maximum values were small for LDH (1.6-fold) and TNFα (1.4-fold), moderate for GLU (2.4-fold), and largest for H_2_O_2_ (3.1-fold). However, as H_2_O_2_ measurements are carried out after 90 min of particle exposure, this part of the test is more sensitive to incomplete gravitational settling of the particles. Of note, the rankings of the time constants for gravitational settling and of apparent EC50 were completely identical (ZrO_2_-TODS < ZrO_2_-PGA < ZrO_2_-APTS < ZrO_2_-Acryl), suggesting that different amounts of H_2_O_2_ primarily reflect the obvious difference in particle exposure to cells. However, we cannot rule out that, e.g., ZrO_2_-Acryl, which induced the steepest slope of dose-dependent H_2_O_2_ release from NR8383 cells, elicits specific effects different from those of ZrO_2_-TODS, ZrO_2_-PGA and ZrO_2_-APTS. Given the pro-oxidative potential of uncoated ZrO_2_ NPs [13], it also appears possible that pro-oxidative structures of the ZrO_2_ NPs surface are incompletely shielded by acrylate residues. With respect to induction of glucuronidase, which was not impaired by differences in particle dynamics, ZrO_2_–Acryl was more effective than other surface modifications, suggesting an activating or modifying effect of acrylate or other residues. In line with this assumption, the surface chemistry of polyacrylate beads (600 nm) has been shown to specifically influence macrophage TNFα production and differentiation after injection of surface-modified particles [30]. Further experiments directly comparing coated and non-coated NPs need to be designed to shed more light on these effects and the underlying mechanisms.

### 3.2. In Vivo Studies on the Rat Lung

Apart from the mere description of in vitro effects, we derived reasonable doses for further in vivo testing in the rat lung. It was a striking finding that extrapolation from no-effect levels in vitro led to a reasonable low-dose estimation for intratracheal administration (0.6 mg/lung), which in fact elicited no adverse effects after three and 21 days. A previous shortterm inhalation study had shown that a slightly higher lung burden of 0.693 mg ZrO_2_-TODS (deposited over five days upon air concentration of 50 mg/m^3^) also had no effect on the rat lung [9], thus confirming the results of the macrophage-based dose estimation. However, both ZrO_2_-TODS and ZrO_2_-Acryl had largely equivalent dose-dependent effects consisting of elevated alveolar macrophage and PMN counts, which reversed after 21 days, except for the highest dose (2.4 mg/lung). Since the particle distribution patterns, the dominant uptake of ZrO_2_ NPs by alveolar macrophages, and the overall lung appearance were nearly identical for ZrO_2_-TODS and ZrO_2_-Acryl (see Figure 5 and Figure 6), the study gave no major hints of the influence of the different particle surfaces on lung toxicity. The only difference was the slightly increased total protein and fibronectin concentration in BALF upon ZrO_2_-TODS, which was not fully restored after 21 days and may reflect the higher cytotoxicity of ZrO_2_-TODS over ZrO_2_-Acryl. 

Interestingly, we were also able to demonstrate uptake of ZrO_2_-TODS and ZrO_2_-Acryl by neutrophilic granulocytes (PMN) both in lung sections and in BALF cytospin preparations (Figure 6 and Figure 8). Uptake of particles and/or nanoparticles by these cells is a controversial and often neglected issue, although PMN are known for their ability to ingest particles secondary to IgG-complement- or serum coating [31,32], whereas mere coating with albumin appears to have a comparably low effect [31]. Although HSI microscopy was not applicable to unstained lung tissue sections due to similarly light-scattering tissue material indistinguishable from ZrO_2_ NPs, application of this method to stained cytospin preparations proved highly useful in showing that particles in PMN and alveolar macrophages share the same quality, thus confirming the in vivo observation. 

The occurrence of ZrO_2_ TODS and ZrO_2_-Acryl NPs in PMN demands careful interpretation and may be due to experimental conditions: Firstly we inspected the high concentration step only (2.4 mg/lung), which certainly is at the border of a temporary overload and, as such, elicited high numbers of PMN in BALF, increasing the chance to identify NPs-laden PMN. Secondly, in our study on rats the preparation of NPs dispersions with such a high concentration (4.8 mg/mL) from acid and basic stock solutions required a pre-coating of NPs with proteins to prevent agglomeration [33]. Rat albumin was chosen because it is contained in the lung lining fluid and can contribute to the protein corona of administered nanoparticles (own unpublished results). 

The conclusion from this part of the study is that the small difference found in vitro for ZrO_2_-TODS and ZrO_2_-Acryl is reflected by the administration study results on rats, although in an attenuated manner. As this attenuation may have been caused at least in part by the albumin pre-coating of ZrO_2_-NPs, results obtained with a lower dose of uncoated NPs in mice are of interest.

### 3.3. In Vivo Studies of Healthy and Allergic Mice

In these experiments one single dose of all ZrO_2_ NPs was selected such that low, though significant, effects were induced, as required for a comparison of particle properties. Using a factor of 10 to approximate the rat/mice body weight ratio and assuming that 1 mg/rat lung would be an adequate concentration led us to apply 100 μg ZrO_2_ NPs per mouse lung. In contrast to the rat study, here we used no further protein coating, a circumstance that led to slightly larger particles in the administration fluid (Figure S3). Nevertheless, the range of effects was as expected and similar to rat experiments as we observed differentially increased numbers of alveolar macrophages, PMN, lymphocytes, and eosinophils in BALF (Figure 10). Again, ZrO_2_-TODS caused the largest effects on PMN counts and because the difference between ZrO_2_-TODS and ZrO_2_-Acryl was larger than observed in the rat, results in the mouse were closer to what was found in the in vitro study. As outlined above, this may be due to the uncoated, i.e., protein-free NPs surface. 

Interestingly, ZrO_2_-PGA was the only particle type to significantly increase lymphocyte and eosinophil numbers in BALF; this finding was even more pronounced in allergic animals. Especially for the allergic mice, this finding strongly deviates from what was found in vitro, although ZrO_2_-PGA was more effective in eliciting TNFα and H_2_O_2_ release from NR8383 alveolar macrophages than other particles. Clearly, the macrophage model, which primarily aims to predict NPs-caused inflammation, does not take into account lung allergic reactions.

The further comparison between non-sensitized and OVA-sensitized allergic animals revealed that ZrO_2_ NPs’ effects on alveolar macrophages, lymphocytes, and eosinophils, but not on PMN, were aggravated in allergic mice. This finding is in line with previous studies showing that allergic lung inflammation is a susceptibility factor for the effect of particle exposure, since alterations in particle deposition caused by allergen sensitization and particle-induced oxidative stress were demonstrated to play a critical role in allergic mice [20,21,34,35]. Since the read-out of the allergic model in this study was chosen to occur five days after NPs administration and OVA challenge, we missed the early neutrophil peak but could evaluate the late inflammatory cell response in the BALF, consisting of macrophages, lymphocytes, and eosinophils.

PGA, especially, appears to have pro-allergic effects. Although PGA is considered to be highly biocompatible even when delivered directly into the blood compartment and biodegradable [16,17,18], it was recently shown that its large available surface area leads to significant membrane interactions and is able to induce macrophage activation [36]. As we tested the ability of different surface modifications to mitigate the pro-inflammatory and immunomodulatory effects of SiO_2_-NPs in a similar model of lung allergic inflammation, we demonstrated that PGA-coated NPs exert a strong adjuvant effect, augmenting the type 2 helper cell (Th2) pro-inflammatory milieu caused by uncoated SiO_2_ particles [10]. Here, we confirm our previous results, showing an increased inflammatory recruitment following intratracheal administration of PGA-coated ZrO_2_ NPs in allergic animals. The question as to whether PGA is useful as a coating agent needs to be considered carefully, especially when allergic individuals are included in targeted NPs applications. 

## 4. Materials and Methods 

### 4.1. Particle Characterization 

Main particle characteristics and the structure of the chemical substances bound to the surface are shown in Table 1. Notably, all particles were diluted from well-dispersed stock suspensions (10% *w*/*w*), previously characterized by the NanoGEM consortium [23,24]. In brief, all ZrO_2_ NPs had a primary size of 9–10 nm (Table S1) and formed loose aggregates, as shown by transmission electron microscopy for the TODS and Acryl-coated ZrO_2_ NPs (Figure 1). The BET size was 117 m^2^/g. Particles were negatively charged within their basic stock solutions, with zeta potentials ranging from −20.5 to −29.2 mV (Table 1). In a pH 7.4 environment, zeta potentials were −39 mV (ZrO_2_-Acryl), −0.5 mV (ZrO_2_-TODS), 3.9 mV (ZrO_2_-APTS), and −7.8 mV (ZrO_2_-PGA). 

### 4.2. In Vitro Experiments

The rat alveolar macrophage cell line NR8383 was used for all experiments. Cells were cultured in 175 cm^2^ culture flasks in F-12K medium (Biochrom GmbH, Berlin, Germany) supplemented with 15% heat-inactivated standardized fetal calf serum at 37 °C and 5% CO_2_. Experiments with ZrO_2_ NPs were performed essentially as described [8]. Cells were incubated with increasing concentrations of particles in serum-free F-12K medium. Therefore, stock solutions with a nominal concentration of 10% (*w*/*w*) were diluted to a working concentration of 180 μg/mL, vortexed, and ultrasonicated with a probe adjusted to 50 W (VibraCell^TM^, Sonics & Materials, Danbury, CT, USA) for 10 s before they were further diluted in doubling steps. As gravimetric measurements later on revealed a deviation in the particle concentrations of some of the ZrO_2_ NPs stock solutions, the concentrations that had been applied to cells were re-calculated and curves were fitted accordingly (see Figure 4). All assays were run in 96-well plates (with 3 × 10^5^ cells per well) and repeated three times. Vehicle-treated cells were used as negative controls. Cells were incubated with increasing concentrations of ZrO_2_ NPs for 16 h. Supernatants were harvested, centrifuged (10 min at 200 g), and each supernatant was assayed for lactate dehydrogenase activity (LDH), glucuronidase activity (GLU), and bioactive TNF-α LDH was measured with the Cytotoxicity Detection Kit (Roche, Germany) and GLU was measured photometrically using *p*-nitrophenyl-β-d-glucuronide as a substrate. Both enzyme activities were expressed as % of the positive control value, which was obtained by adding 0.1% Triton X-100 to an equal number of untreated cells. Bioactive TNF-α was measured indirectly via induction of apoptosis/necrosis in L-929 fibroblasts in the presence of actinomycin D and was expressed as % killing activity. Release of H_2_O_2_ was measured 90 min after the addition of particles, which were suspended in a Krebs–Ringer buffer containing 2 mmol/L glucose (KRPG). H_2_O_2_ concentration was determined quantitatively using resorufin as a detection reagent in the presence of horseradish peroxidase. In all assays we used cell-free wells, which were processed in the same way to exclude particle interference.

### 4.3. Preparation of Particle Suspension for Administration Experiments in Rats

Since the pH values of the ZrO_2_-TODS or ZrO_2_-Acryl stock suspensions (nominal concentration 10% *w*/*w*) were 4 or 10, respectively, the administration fluids were buffered with a bicarbonate buffer system as it adopts a physiologic pH value in the lung due to the alveolar partial pressure of CO_2_. In the first step, one volume of the ZrO_2_-TODS or ZrO_2_-Acryl stock suspension was mixed with one volume of 10% rat serum albumin (Sigma-Aldrich GmbH, Darmstadt, Germany) in distilled H_2_O to prevent NPs agglomeration. Unbound protein was then separated from NPs by gel filtration. Suspensions were passed through small columns (2 cm × 9 cm) filled with CL6B Sephadex (Sigma-Aldrich GmbH, Darmstadt, Germany) previously equilibrated with 25 mM NaHCO_3_ (ZrO_2_-Acryl) or H_2_O (ZrO_2_-TODS). Particle-containing fractions were pooled and their particle content quantified gravimetrically. The final fluid was adjusted to 4.8 mg of either particle per mL and contained 25 mM NaHCO_3_. The albumin content of administered fractions was estimated by the Lowry method with rat albumin as a standard and amounted to 7.7 ± 1.1% and 15.5 ± 3.2% of the total particle mass for ZrO_2_-TODS and ZrO_2_-Acryl, respectively. Gassing of the final fluid with 5% CO_2_ resulted in pH values of 7.26 (ZrO_2_-TODS) and 7.73 (ZrO_2_-Acryl). Particle size distribution was tested by optical tracking analysis using a NanoSight LM10 instrument equipped with a green laser (532 nm), an Andor CCD camera, and NTA software 2.1 (Malvern Instruments GmbH, Herrenberg, Germany). Mean particle sizes were 80 and 71 nm for ZrO_2_-TODS and ZrO_2_-Acryl, respectively (Figure S2).

To generate surface-labeled fluorescent particles, the aqueous albumin-coated suspension was reacted with 20 μg/mL TexasRed^®^ succinimidyl ester (TxR, Invitrogen, Molecular Probes, Eugene, OR, USA) at room temperature for 30 min, then passed through a gel filtration column as described. Eluted fluorescent particle fractions were identified with a plate reader (Tecan Infinite F200 Pro, Männedorf, Switzerland), pooled, and adjusted to 4.8 mg/mL with 25 mM NaHCO_3_ after gravimetric measurement. 

### 4.4. Preparation of Particle Suspension for Administration Experiments in Mice

To avoid protein interactions in experiments with allergic animals and because a low dose of ZrO_2_ NPs was given in these experiments we used a different protocol to prepare the dosing fluid. Stock solutions (10% *w*/*w*) of ZrO_2_-TODS, ZrO_2_-APTS, and ZrO_2_-Acryl were diluted approximately 50-fold with 0.9% NaCl; ZrO_2_-PGA was diluted with ultra-pure H_2_O (aqua ad iniectabilia) as it precipitates immediately in 0.9% NaCl. All suspensions were then buffered with PBS (200–400 μL) yielding pH values of 6.5–7.0, and adjusted to contain 100 μg ZrO_2_ NPs in approximately 50 μL of solution. The particle size achieved with this protocol was somewhat larger than in the presence of protein and is shown in Figure S3. Control solutions (CTL) were obtained by hard sedimentation (28,000 g, 15 h) of the ZrO_2_ NPs, which reduced the particle concentration by 95–98%. For sensitized animals, only supernatant of ZrO_2_-PGA was used. 

### 4.5. Animal Experiments 

#### 4.5.1. Experiments with Rats

Animal experiments were conducted at the animal facility of the University Clinics of Essen, Germany and ethically approved by LANUV (Dortmund, Germany, No. 84-02.04.2022.A157). Female Wistar rats, strain WU, weighing 200–250 g (Charles River Laboratories, Sulzfeld, Germany), were maintained in a 12 h lights-on lights-off environment. Food and water were provided ad libitum. Animals (*n* = 5 per group) were briefly anesthetized with isoflurane and received 500 μL of instillation fluid containing 0.6, 1.2, or 2.4 mg (*w*/*v*) of either ZrO_2_-TODS or ZrO_2_-Acryl NPs. Control animals received vehicle only (25 mM NaHCO3, pH 7.4). In addition, we delivered albumin-coated and Texas Red-labeled ZrO_2_-TODS (*n* = 4), and ZrO_2_-Acryl (*n* = 4) at a concentration of 1.2 mg/lung to investigate particle distribution in the lung after 3 h and three days. Intratracheal administrations were carried out under visual control using a Penn Century Microsprayer and rats recovered from this procedure within less than 30 min. After three and 21 days, animals were deeply anaesthetized with a mixture of ketamine and xylazine and sacrificed by bleeding from the *Aorta descendens*. A cannula was inserted into the trachea and, while the left bronchus was transiently closed with a Diefenbach clamp, the right lung was lavaged five times with 3 mL 0.9% NaCl, yielding ca. 14 mL of broncho-alveolar lavage fluid (BALF) per animal. After the right bronchus was clamped, the left lung was inflated with 3 mL Cryomatrix (Thermo Shandon Ltd., Runcorn, UK), resected, flash-frozen in liquid nitrogen, and stored at −80 °C for histologic studies. Lungs dosed with fluorescent particles were filled with 5 mL Cryomatrix for histology only.

#### 4.5.2. Experiments with Mice

Female, 6–10-week-old BALB/c mice were obtained from Charles River (Sulzfeld, Germany), housed under specific pathogen free conditions in individually ventilated cages (VentiRack, Biozone, Margate, UK), and fed a standard diet and water ad libitum. The study was conducted under federal guidelines for the use and cares of laboratory animals and was approved by the Government of the District of Upper Bavaria (Approval No. 55.2-1-54-2532-156-12) and the Animal Care and Use Committee of the Helmholtz Center, Munich. 

In order to evaluate the adjuvant effects of ZrO_2_ NPs, a protocol of mild allergic inflammation of the lung was employed. Mice were sensitized by repetitive intraperitoneal injections of 1 μg OVA (grade VI, Sigma-Aldrich Chemie GmbH, Taufkirchen, Germany)/Alum (Pierce, 2.5 mg, Sigma-Aldrich Chemie GmbH, Taufkirchen, Germany) in phosphate-buffered saline (PBS) on days 0, 7, 14, 28, and 42. Blood samples were taken before and after sensitization in order to control for increases in OVA-specific IgE and IgG1 titers at the end of the sensitization protocol. The antibodies were measured in plasma samples by ELISA, as described previously [20]. On day 52 the mice received a single intratracheal administration of 100 μg ZrO_2_ NPs or CTL prior to OVA challenge, which was delivered for 20 min by a Pari-Boy nebulizer (Pari, Starnberg, Germany). The animals were sacrificed five days after OVA challenge.

### 4.6. Analyses of Broncho-Alveolar Lavage Fluid (BALF)

Cells from pooled rat BALF preparations were collected at the bottom of a centrifuge vial (200 g, 4 °C, 10 min). The supernatant was centrifuged again and the final supernatant was used for protein determination according to the Lowry method, using bovine serum albumin as a standard. Fibronectin was detected by a specific ELISA [37]. Final numbers of cells were determined with a Coulter counter (model Z2, Beckman Coulter GmbH, Krefeld, Germany) and the proportion of dead cells was determined by trypan blue testing. Differential cell counting was carried out with cytospin preparations stained with May-Grünewald and Giemsa dyes. At least 400 cells per animal were evaluated under the light microscope.

Mouse airways were lavaged five times with 0.8 mL PBS. After centrifugation (1100 rpm, 10 min), the viability and yield of BALF cells were quantified via trypan blue (Serva, Germany) exclusion in a hemocytometer. Differential BALF cell count was performed on cytospins as described for the rat BALF. 

### 4.7. Immunocytochemistry, Fluorescence Microscopy, and Hyperspectral Imaging 

Transverse sections (7 μm) were cut from the hilar region of the left lung with a cryo-microtome (Microtome Cryostsat HM 500, MICROM International GmbH, Walldorf, Germany). Sections were dried onto glass slides and stored at −20 °C. To stain for alveolar macrophages or neutrophilic granulocytes (PMN), frozen sections were post-fixed with 4% phosphate-buffered formaldehyde for 15 min, or methanol/acetone 1:1 mixture for 10 min, respectively. Sections were rinsed in phosphate-buffered saline (PBS) and non-specific binding sites were blocked with 3% bovine serum albumin (BSA, fraction V, Serva). An anti-CD68 antibody (AbDSerotec, diluted 1:100 in PBS, 1% BSA) or an anti-PMN antibody (Antibodies online, no. ABIN636445, diluted 1:5000 in PBS, 1% BSA) was used to label alveolar macrophages and PMN, respectively. Bound anti-CD68 antibody was detected with a selective secondary anti-mouse IgG antibody conjugated to Alexa Fluor^®^488 (Cell Signaling Technology, Leiden, The Netherlands, diluted 1:20,000); anti-PMN antibody was labeled with anti-rabbit Alexa Fluor^®^555. Sections were rinsed thoroughly in PBS and cover slipped with Roti^®^-Mount FluorCare (Carl Roth, Karlsruhe, Germany) which contained 4′,6-diamidin-2-phenylindol (DAPI) to stain for the DNA of cell nuclei. To visualize the distribution of fluorescently labeled ZrO_2_ NPs in the lung, sections were post-fixed, washed, and cover slipped as above. Micrographs were taken with a Nikon DVC CCD camera and Nikon Lucia 4.3 software (Nikon GmbH, Düsseldorf, Germany) mounted on a Olympus IX51 fluorescence microscope (Olympus Deutschland GmbH, Hamburg, Germany) equipped with filter sets for DAPI, Texas Red, and fluorescein to collect blue, red, and green fluorescence, respectively. Unstained and immunostained sections were also viewed with a Olympus BX50 microscope equipped with an enhanced dark field condenser and a hyperspectral imaging device (CytoViva Inc., Auburn, AL, USA). Images were analyzed with a CytoViva plugin to ENVI 4.0 Software licensed to and distributed by CytoViva Inc. (CytoViva Inc., Auburn, AL, USA).

### 4.8. Statistics 

In vitro data were generated in triplicate and at least three independent repetitions were carried out. Values for each concentration were compared to non-treated controls by two-way ANOVA and Bonferroni multiple comparisons test. EC50 concentration values were calculated from nonlinear regression curves calculated by GraphPad Prism 6.01, plotting the logarithmic concentrations of NPs concentrations against effects (in % control); quality of fit was indicated by R^2^ values. For in vivo testing (five animals per group) all data are expressed as mean ± standard deviation (SD). BALF data from rats were compared pair-wise to the control group by one-way ANOVA and post hoc Dunnett’s multiple comparison test. BALF data from mice were compared by one-way ANOVA followed by the Kruskal-Wallis test. For all experiments, *p* ≤ 0.05 was considered significant.

## 5. Conclusions

Investigations show that surface functionalization of ZrO_2_ nanoparticles with different organic molecules such as tetraoxadecanoic acid, acrylic acid, aminopropyl silane, or polyethylene glycol cause minor differences in the particles’ in vitro and in vivo toxicity, even at comparably high concentrations. Allergic animals, however, are more susceptible compared to healthy animals and respond to single coatings in a more specific manner that is not evident from ordinary rodent studies and from cell culture testing with alveolar macrophages. Of note, a previous attempt to characterize and cluster the NanoGEM nanomaterials via concentration-dependent surface adsorption found many similarities between ZrO_2_-TODS, ZrO_2_-Acryl, and ZrO_2_-APTS, whereas ZrO_2_-PGA was separated especially by a Langmuir model based on a low concentration approximation [38]. Apparently this classification parallels the overall result from our study with allergic mice and may, therefore, open up new possibilities for identifying the surface elements of nanoparticles involved in allergic responses. In any case, care should be taken in transferring results from in vitro and in vivo studies carried out on rodents to human individuals; polyethylene glycol, especially, deserves special attention as a coating agent.

## Figures and Tables

**Table 1:** EC50 values of in vitro effects on NR8383 cells.

Assayed Parameter	ZrO_2_–TODS	ZrO_2_–Acryl	ZrO_2_–PGA	ZrO_2_APTS
LDH	43.7 (0.89) ^1^	66.5 (0.85)	67.9 (0.85)	68.5 (0.91)
GLU	68.5 (0.87)	143.43 (0.83)	100.0 (0.66)	162.8 (0.88)
H_2_O_2_	31.7 (0.8)	97.7 (0.91)	n.d.	n.d.
TNFα	53.8 (0.83)	61.6 (0.91)	51.1 (0.85)	75.5 (0.97)

^1^ Numbers give concentration values in μg/mL; *R*^2^ values are shown in brackets. n.d. values could not be calculated.

## Supplementary Materials

The following are available online at http://www.mdpi.com/2079-4991/7/9/280/s1, Figure S1: In vitro effects of supernatants generated from particle ZrO_2_ NPs preparations, Figure S2: Size distribution of rat serum albumin-coated ZrO_2_ particles as prepared for intratracheal administration in rats, Figure S3: Size distribution of ZrO_2_ nanoparticles dispersed in 0.9% NaCl/PBS as used for an administration study in ovalbumin-sensitized mice. Table S1: Characteristics of zirconia nanoparticles as published by the NanoGEM consortium.
