# Supplementary material for: Differential Effects of Surface-Functionalized Zirconium Oxide Nanoparticles on Alveolar Macrophages, Rat Lung, and a Mouse Allergy Model
Source: Nanomaterials (Basel). 2017 Sep 19;7(9):280. doi: 10.3390/nano7090280 (PMC5618391; doi:10.3390/nano7090280)
Supplement: Supplementary file 1 [file nanomaterials-07-00280-s001.pdf]

Supplementary Information

Differential Effects of Surface-Functionalized Zirconium Oxide Nanoparticles on Alveolar Macrophages, Rat Lung, and a Mouse Allergy Model

Antje Vennemann [1], Francesca Alessandrini [2] and Martin Wiemann [1,*]

[1] IBE R&D Institute for Lung Health gGmbH, Mendelstr. 11, 48149 Münster, Germany; vennemann@ibe-ms.de

[2] Center of Allergy and Environment (ZAUM), Technical University of Munich and Helmholtz Center Munich, Member of the German Center for Lung Research (DZL), Ingolstädter Landstr. 1, 85764 Neuherberg, Germany; franci@helmholtz-muenchen.de

* Correspondence: martin.wiemann@ibe-ms.de; Tel.: +49-251-9802340

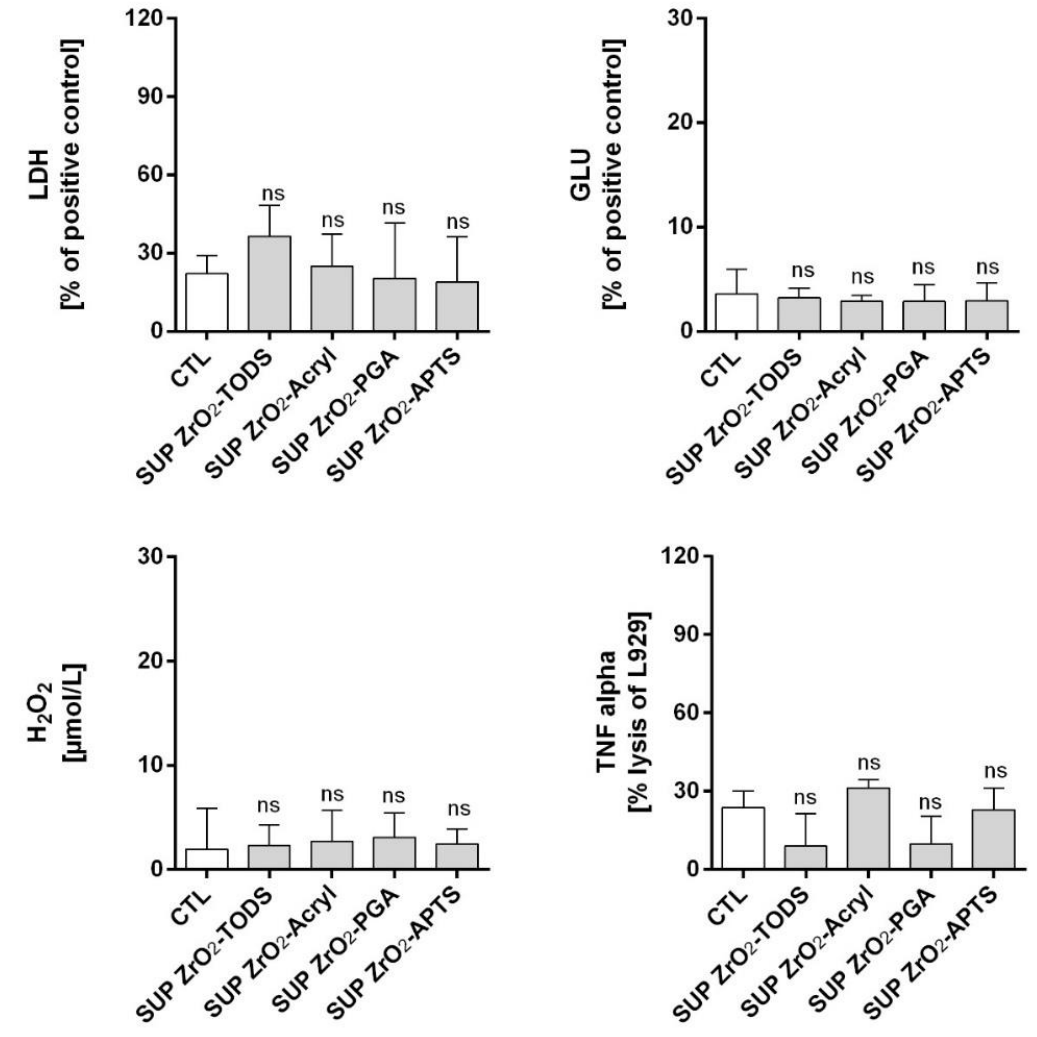

Figure S1. In vitro effects of supernatants generated from particle ZrO_2 NPs preparations. Particle stock suspensions of ZrO_2-TODS, ZrO_2-Acryl, ZrO_2-PGA, and ZrO_2-APTS were subjected to centrifugation (28,000 g, 8 h) to obtain particle-free supernatants (SUP). Volumes equivalent to the 180 µg/mL concentration (c.f. Figure 4) were then diluted in F-12K medium or KRPG buffer and applied to NR8383 macrophages. (ns) not significant, (CTL) untreated control cells.

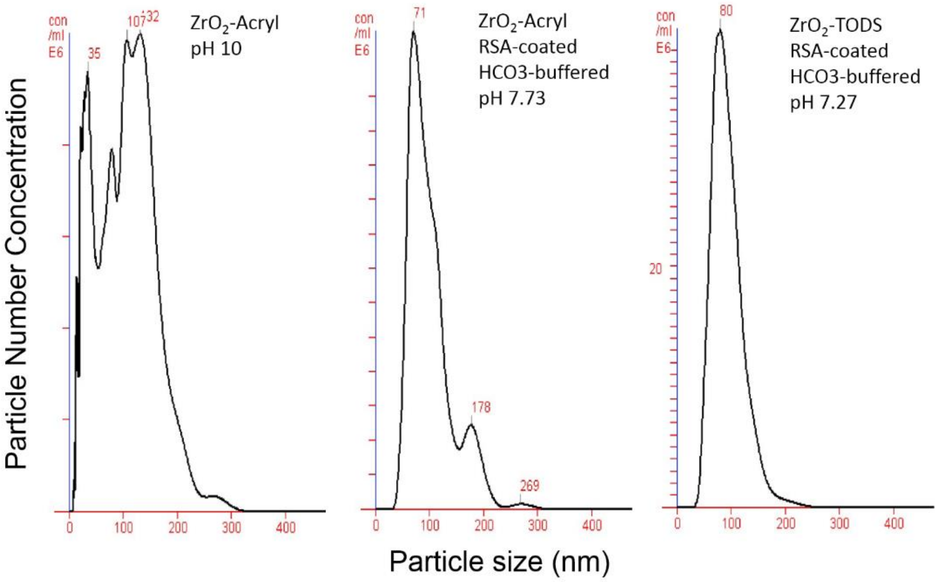

Figure S2. Size distribution of rat serum albumin-coated ZrO_2 particles as prepared for intratracheal administration in rats. Particles were buffered with 25 mM HCO_3^- and 5% CO_2. Measurements were carried out with optical particle tracking and NanoSight Software NTA 2.2 (Minton Park Amesbury, Wiltshire SP4 7RT, UK).

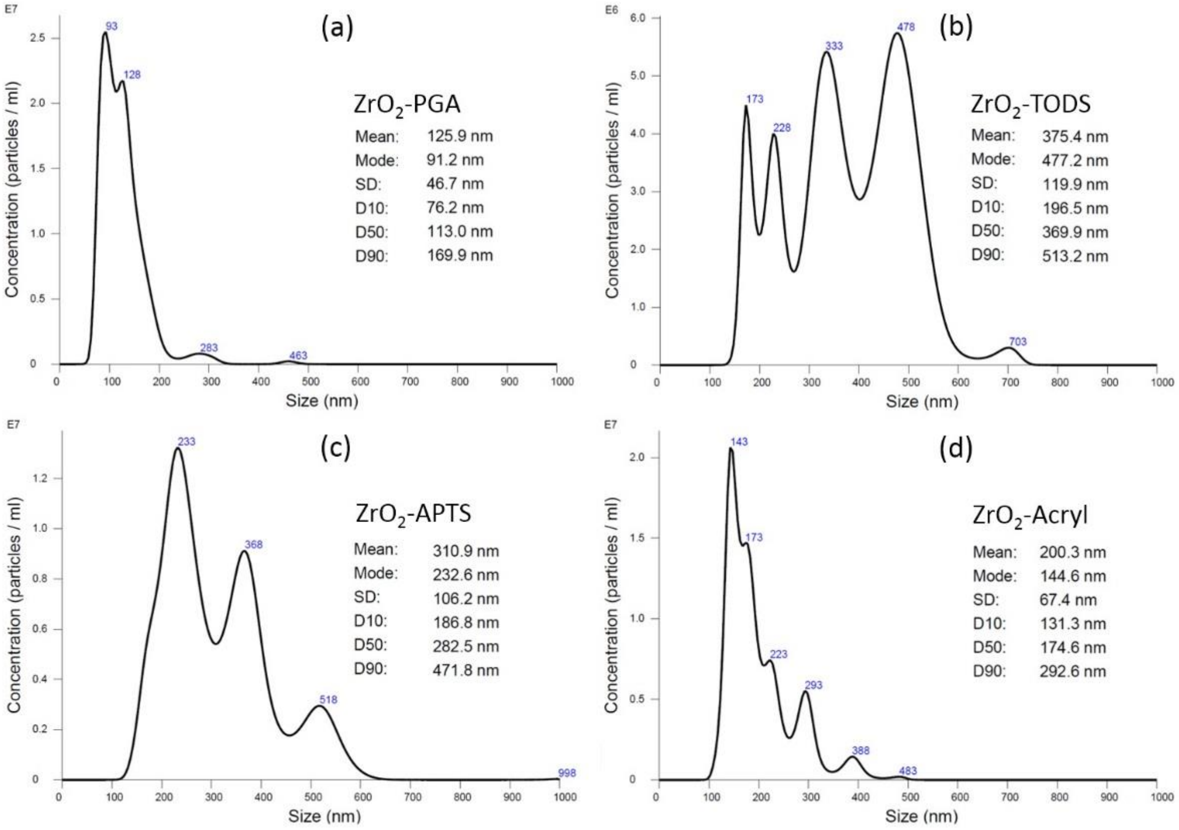

Figure S3. Size distribution of ZrO_2 nanoparticles dispersed in 0.9% NaCl/PBS as used for an administration study in ovalbumin-sensitized mice. (**a**) ZrO_2-PGA, (**b**) ZrO_2-TODS, (**c**) ZrO_2-APTS, and (**d**) ZrO_2-Acryl. Measurements were carried out with optical particle tracking and NanoSight Software NTA 3.1 (Minton Park Amesbury, Wiltshire SP4 7RT, UK).

Table S1. Characteristics of zirconia nanoparticles as published by the NanoGEM consortium. Data are taken from references [23,24] cited in the main text.

	**ZrO_2-Acryl**	**ZrO_2-PGA**	**ZrO_2-APTS**	**ZrO_2-TODS**
Organic residue	polyacrylic acid	polyethylene glycol (600 g/mol)	aminopropyltrimethoxy silane	trioxadecanoic acid
Particle size TEM [nm}	9	9	10	9
BET surface	117	117	105	117
Zeta potential (mV)	-29.2	-25.2	-20.5	-25.1
Structure	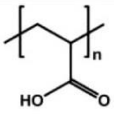	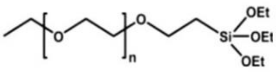	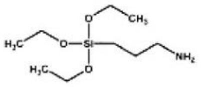	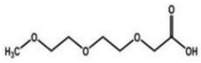
Formula	-	HOOC-CH_2-(OC_2H_4)n-O-CH_2-COOH	H_2N-C_3H_6-$Si(OCH_3)_3$ $SiC_6H_{15}O_3N$	HOOC-CH_2-O-C_2H_4-O-C_2H_2-O-CH_3 $C_7O_5H_{14}$
